# Exploring *Echinacea angustifolia* for anti-viral compounds against Zika virus RNA-dependent RNA polymerase: a computational study

**DOI:** 10.1038/s41598-025-88481-8

**Published:** 2025-02-03

**Authors:** Mai M. El-Daly, Leena H. Bajrai, Thamir A. Alandijany, Isra M. Alsaady, Hattan S. Gattan, Meshari M. Alhamdan, Vivek Dhar Dwivedi, Esam I. Azhar

**Affiliations:** 1 Special Infectious Agents Unit – BSL3, King Fahd Medical Research Center, King Abdulaziz University, 21362 Jeddah, Saudi Arabia; 2 Department of Medical Laboratory Sciences, Faculty of Applied Medical Sciences, King Abdulaziz University, 21362 Jeddah, Saudi Arabia; 3 Biochemistry Department, Faculty of Sciences, King Abdulaziz University, 21362 Jeddah, Saudi Arabia; 4 Family Medicine Department, Faculty of Medicine, King Abdulaziz University, 21589 Jeddah, Saudi Arabia; 5 Center for Global Health Research, Saveetha Institute of Medical and Technical Sciences, Saveetha Medical College and Hospitals, Saveetha University, Chennai, India; 6 Bioinformatics Research Division, Quanta Calculus, Greater Noida, India

**Keywords:** Zika virus, RNA-dependent RNA polymerase (RDRP), *Echinacea angustifolia*, MD simulation, Virtual drug screening, Virtual screening

## Abstract

The Zika virus (ZIKV), a member of the Flaviviridae family, has caused multiple widespread outbreaks, posing significant challenges to global health. This study explores the potential of compounds from *Echinacea angustifolia* (*E. angustifolia*) to inhibit the activity of ZIKV’s RNA-dependent RNA polymerase (RDRP), a key enzyme in the viral replication process and an ideal candidate for antiviral therapy. Utilizing computational techniques, we conducted a thorough virtual examination using the MTi-OpenScreen tool to identify potential RDRP inhibitors among *E. angustifolia* compounds. The top four compounds were further examined through re-docking procedures. To assess the robustness and effectiveness of these interactions, we performed molecular dynamics simulations along with calculations of the binding free energy and PCA analysis. This investigation highlighted four naturally occurring compounds, viz., Echinacoside, Rutin, Echinacin, and Cynaroside, demonstrating a notable affinity for binding to the allosteric site of ZIKV RDRP. These compounds showed strong hydrogen bond formation with crucial residues of the RDRP and presented favorable binding free energies. Our research sheds light on the viability of these *E. angustifolia* compounds as ZIKV RDRP inhibitors, laying a foundation for further experimental research in developing novel antiviral treatments against ZIKV infections.

## Introduction

The Zika virus (ZIKV), belonging to the Flaviviridae family, is a pathogen transmitted primarily by Aedes mosquitoes, prevalent in tropical and subtropical areas^1,2^. Besides mosquito bites, ZIKV can spread through sexual transmission, blood transfusions, and from pregnant women to their unborn children^3–6^. While most people infected with ZIKV show no symptoms, those who do may experience mild conditions like fever, rash, joint pain, and conjunctivitis. In some cases, especially among pregnant women, ZIKV can cause severe neurological disorders such as microcephaly and Guillain-Barré syndrome^1,7^. Treatment for ZIKV is currently limited to symptom management and supportive care. Preventive measures like using insect repellent, wearing protective clothes, and destroying mosquito breeding grounds are vital in curtailing the spread of the virus. The global health threat posed by ZIKV underscores the necessity for comprehensive research into its transmission, pathology, and potential treatments. A significant focus is on finding and confirming effective drug targets against ZIKV. One such target is the RNA-dependent RNA polymerase (RDRP), a key enzyme in the virus’s replication cycle^8–10^. The identification of inhibitors that specifically target the ZIKV RDRP could provide a novel therapeutic approach to combat the infection. Medicinal plants have been used for centuries to treat various ailments, including viral infections^11–13^. These plants are rich in bioactive components, offering a treasure trove for the development of antiviral pharmaceuticals^14–17^. Among these, E. angustifolia stands out for its renowned immune-enhancing qualities, a staple in the realm of traditional healing practices^18^. This plant has garnered attention in scientific circles for its efficacy against various viruses, such as Influenza, Herpes simplex, Japanese encephalitis, and even the recent SARS-CoV-2^19–22^. The specific constituents of Echinacea that impart its antiviral capabilities remain an area of active research, though compounds like polysaccharides, alkamides, and caffeic acid derivatives are known to play a significant role^23^ .

This study delves into the potential of E. angustifolia as a natural reservoir of compounds effective against the Zika virus (ZIKV). Our primary objective is to isolate potential inhibitors targeting the ZIKV RDRP and to unravel their inhibitory mechanisms via detailed molecular dynamics simulations. This exploration is not just about identifying new inhibitors; it’s about deepening our understanding of how these natural compounds interact at a molecular level with the ZIKV, potentially paving the way for novel treatment strategies.

Moreover, this research extends beyond the scope of just finding inhibitors. It seeks to contribute to the broader field of phytotherapy, highlighting the untapped potential of plant-based compounds in modern medicine. By investigating E. angustifolia’s components, this study aims to illuminate the intricate ways in which natural substances can interact with viral pathogens, offering insights that could revolutionize how we approach viral infections. The findings from this study could have far-reaching implications, not only providing new avenues for ZIKV treatment but also setting a precedent for the use of phytochemicals in combating other viral diseases. This research, therefore, stands at the forefront of bridging traditional herbal wisdom with cutting-edge scientific inquiry, potentially unlocking new frontiers in antiviral therapy.

## Methodology

### Structure data collection and preparation

The three-dimensional structure of ZIKV RDRP in complex with its inhibitor solved at 1.94 Å resolution was obtained from protein data bank (PDB id: 6LD5)^24,25^. The phytochemical structures of *E. angustifolia* were downloaded from the PubChem database^26^. Preparing the protein structure is an important step before performing docking studies. The preparation of a protein structure was performed in UCSF Chimera using the Dock prep tool, which involved loading the structure, checking for errors, adding missing atoms and residues, removing unwanted molecules, optimizing the structure, and saving the optimized structure as a new PDB file^27^. This preparation protocol ensures the quality and accuracy of the docking results and aids in identifying potential ligands for further study.

### Structure-based screening (SBVS) and re-docking

Virtual screening is a computational technique to predict small molecules’ binding affinity to a protein target. MTiOpenScreen is a web server that offers a user-friendly platform for virtual screening, which was used in this study^28^. The docking grid was generated by selecting the inhibitor molecule binding residues present in the crystal structure of Zika RDRP. The center coordinate was set at 77.36 (x), -3.58 (y), and 13.19 (z) with the grid box size of 25 × 25 × 25. SBVS of *E. angustifolia* phytomolecules using MTiOpenScreen were involved in preparing the protein structure, uploading the structure to MTiOpenScreen, preparing the ligand database, setting up the virtual screening parameters, running the virtual screening, and analyzing the results. The first pose of each 4 selected phytomolecules was taken for re-docking studies using the Autodock vina UCSF Chimera plugin that can provide valuable insights into the interactions between small molecules and protein targets^27,29^. The same docking parameters were set as in the SBVS experiment to validate the screening results.

### MD simulation experiments

MD simulation of Zika RDRP-*E. angustifolia* compounds using free academic Desmond-maestro inter-polarity tool involved in preparing the protein-ligand complex, parameterization of the ligand, solvation, and neutralization, energy minimization, equilibration, production run, and data analysis^30,31^.

Desmond is a widely used software package for molecular dynamics simulation of biomolecules. The following steps were followed to perform MD simulations of the Zika RDRP-*E. angustifolia* compounds complex using Desmond.

#### Preparation of protein-ligand complex

Each protein-ligand complex was prepared in the first step using free academic Desmond-maestro by adding missing atoms and residues, removing water molecules and other ligands, assigning bond orders and hydrogen atoms, and optimizing the protein-ligand complex geometry and energy.

#### System setup for protein-ligand complexes

Setting up a molecular dynamics (MD) system in Desmond involves several steps, including preparing the input files, parameterizing the system, solvating and neutralizing the system, and energy minimization. Each protein-ligand complex was solvated in a box of water molecules (TIP4P) and neutralized by adding counter ions to the simulation box. After system setup for each complex, it was minimized to remove any steric clashes or bad contacts. It was achieved using the Desmond minimization module.

#### Equilibration and production run

The system needs to be equilibrated to reach a stable conformation before the production run. This includes two steps: (a) NVT equilibration, which keeps the temperature constant and allows the water molecules and counter ions to adjust to the system, and (b) NPT equilibration, which keeps the temperature and pressure constant and allows the protein-ligand complex to adjust to the solvent environment. The default parameters in Desmond molecular dynamics module were selected for system equilibration.

After equilibration, each system was subjected to a production run for 200 ns, which is a long MD simulation that allows the protein-ligand complex to explore the conformational space and the ligand-binding pocket. The production run was also performed using the Desmond MD module with default parameters such as time step, temperature, and pressure.

#### Simulation interaction diagram analysis

The MD simulation data were analyzed using the Simulation Interaction Diagram Analysis tool of Desmond. The analysis includes tasks such as trajectory visualization, RMSD and RMSF calculation, and identification of key binding interactions between the Zika RDRP and E. angustifolia compounds.

### MM/GBSA calculation

The MM/GBSA method was employed to calculate the binding free energy of the selected *E. angustifolia* and Zika-RDRP complexes using the prime tool of the Schrodinger suit^32,33^. The last 50 ns trajectories of each complex were taken for analysis. The molecular mechanics energy (EMM) was calculated for 50 ns simulation trajectories at each 1 ns interval using the OPLS-2005 force field. The Generalized Born (GB) solvation energy was calculated using the GB model^34^. The binding free energy (ΔG_Bind_) was calculated using the following equation:

$$\Delta \text{G}_{\text{Bind}} = \Delta \text{G}_{\text{MM}} + \Delta \text{G}_{\text{GB}} + \Delta \text{G}_{\text{SA}}$$

Where ΔG_MM_ is the molecular mechanics energy difference between the protein-ligand complex and the separated protein and ligand, ΔGB_GB_ is the generalized Born solvation energy difference, and ΔG_SA _is the nonpolar solvation energy estimated by the solvent accessible surface area (SASA) of the protein-ligand complex.

### Principal component analysis

Principal component analysis (PCA) analysis offers valuable information about the flexibility and structural changes observed in complex^35^. This helps to understand better how these complexes bind and behave over time, improving our interpretation of their dynamics. PCA was conducted using the Bio3D package in R to investigate the conformational dynamics of the selected phytochemical compounds obtained from *E. angustifolia* and the reference molecule that is docked with ZIKV RDRP after MD simulation^36^. Prior to the PCA analysis, initially, the atomic coordinates of each phytochemical complex were retrieved from their simulation trajectory files. These coordinates were loaded into the Bio3D package, where they were converted into a dcd file format and then loaded into R using the read.pdb function available in the above-mentioned package^36^. The loaded R structure and the initial structure of the complexes generated during the MD simulation were aligned and superimposed using the align function and the fit.xyz function available in the Bio3D package. The generated output trajectories of each complex were further statically calculated using cov.pca function to find the correlation between the atomic deviations obtained in each complex using the covariance matrix calculation method. The values generated after this calculation are represented in eigenvectors, and these eigenvectors are considered as principal components, which are plotted during the PCA analysis. The generated conformers of each protein-ligand complex which are projected in cluster format and these clusters were also analysed for PCA analysis.

### DCCM analysis

The dynamic cross-correlation matrix (DCCM) analysis, performed using the bio3D package in Rstudio, examined the entire molecular dynamics trajectory to evaluate correlated motions between protein residues^36^. By calculating the cross-correlation of atomic displacements, the analysis identifies regions with coordinated or anti-correlated movements, providing insights into protein stability, flexibility, and potential allosteric communication pathways. This analysis is valuable for understanding the dynamic behavior of protein-ligand complexes, revealing structural rigidity and functional motions. The heatmap visualization aids in identifying key regions involved in protein function and interaction, making DCCM crucial for drug discovery and studying protein dynamics.

### Secondary structure analysis

The secondary structure analysis was performed using Visual Molecular Dynamics (VMD) to track changes in the protein’s secondary structural elements, such as alpha helices, beta sheets, and random coils, throughout the molecular dynamics (MD) simulation^37,38^. The Timeline plugin in VMD was used to assign and visualize these structures based on backbone dihedral angles, providing a clear, color-coded representation of their evolution during the simulation. This analysis is crucial for assessing the stability and flexibility of the protein, particularly in understanding how ligand binding or environmental changes affect structural elements. It also offers insights into conformational dynamics and protein folding mechanisms, key for understanding protein function and guiding drug discovery.

### RG-RMSD based FEL

The RG-RMSD-based free energy landscape (FEL) analysis using the Geo-measure plugin in PyMOL was conducted to assess the stability and conformational changes of protein-ligand complexes during molecular dynamics simulations^39^. RMSD was used to track structural deviations, while RG measured the compactness of the complex. The 2D FEL plot, with color-coded energy states, highlighted stable conformations (low-energy minima) and transitional states (high-energy regions). This analysis helps identify the most stable binding conformations, providing insights into ligand stability and protein-ligand interactions. It aids in understanding conformational flexibility and potential allosteric sites, which are key for drug design. By pinpointing regions of low energy and compact structures, the analysis helps select lead compounds with stronger binding affinity and better therapeutic potential.

## Results and discussion

### Virtual screening experiment

**Fig. 1:**
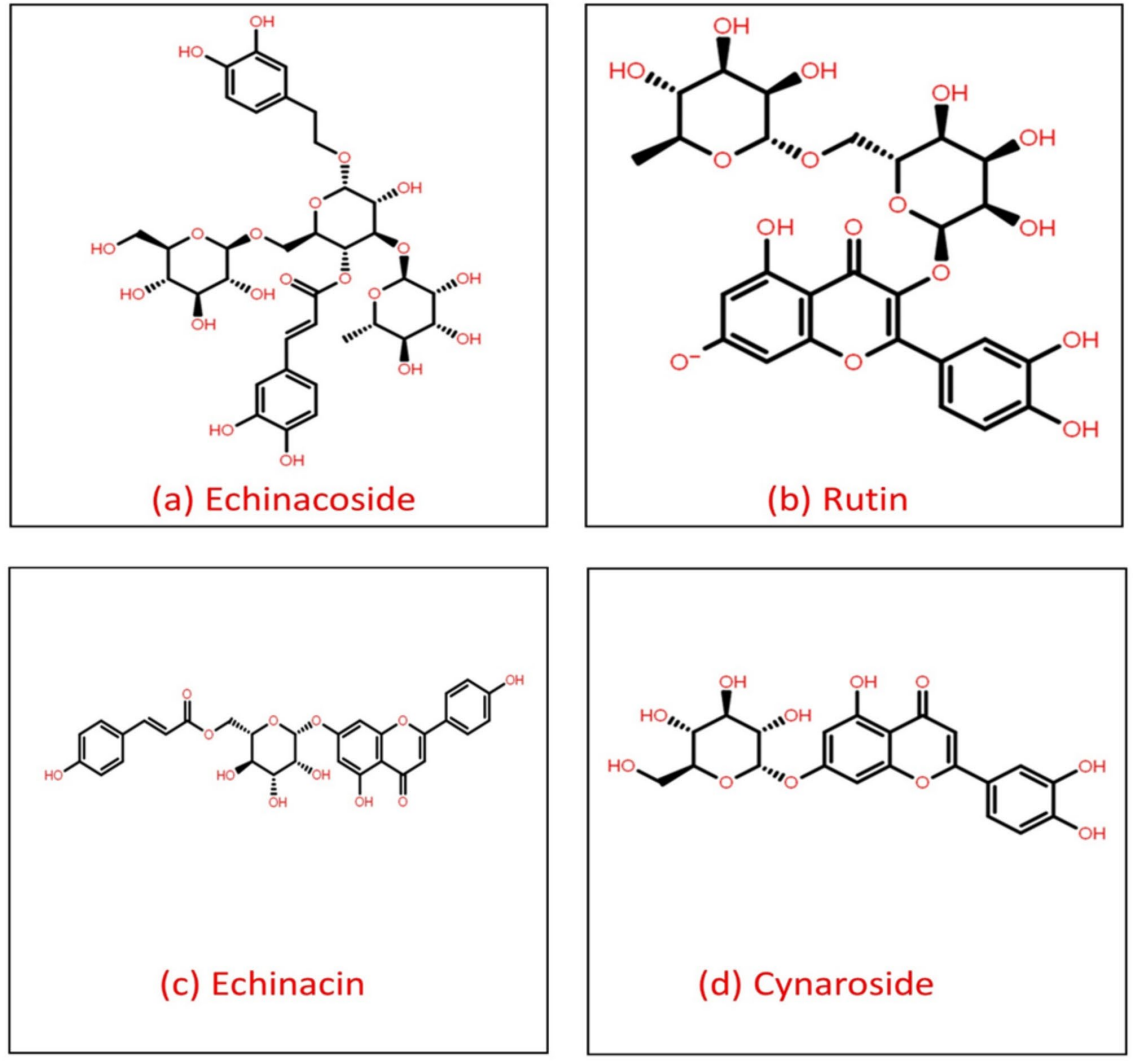
The 2D structure of the top ranked phytochemical compounds selected for further analysis.

To examine the potential of 45 phytomolecules from E. angustifolia against the RNA-dependent RNA polymerase (RDRP) of the Zika virus, we carried out an extensive in-silico investigation. The docking energy of these compounds was determined by employing a method known as virtual screening, which was carried out with the assistance of the MTi-OpenScreen website^28^. The docking energy ranged from −10 to −2 Kcal/mol, which indicated that the binding contacts were favourable. Among the chemicals that were evaluated, Echinacoside, Rutin, Echinacin, and Cynaros revealed very strong docking energies of −10.0, −9.6, −9.4, −9.3 kcal/mol, respectively and robust binding interactions with the RDRP of the Zika virus. Based on these encouraging findings, these compounds were chosen for additional research, drawing attention to their potential as lead candidates in the development of innovative anti-Zika treatments (Fig. 1, Supplementary Table S1).

### Re-docking, and molecular interaction analysis

A re-docking and molecular interaction investigation was carried out to learn more about the binding interactions between Echinacoside, Rutin, Echinacin, and Cynaroside and the Zika virus RDRP. Using the appropriate docking tool (AutoDock vina), the compounds were placed back into the Protein’s active site. According to the re-docking score obtained i.e., −10.1 kcal/mol for Echinacoside, 9.5 kcal/mol for Rutin, 9.4 kcal/mol for Echinacin, and −9.3 kcal/mol for Cynaroside, all these compounds retained their favourable binding within the active site. Also, the reference molecule (G8O-G8L) (Supplementary Fig. 1) displayed an acceptable re-docking score of −9.0 kcal/mol.

**Fig. 2:**
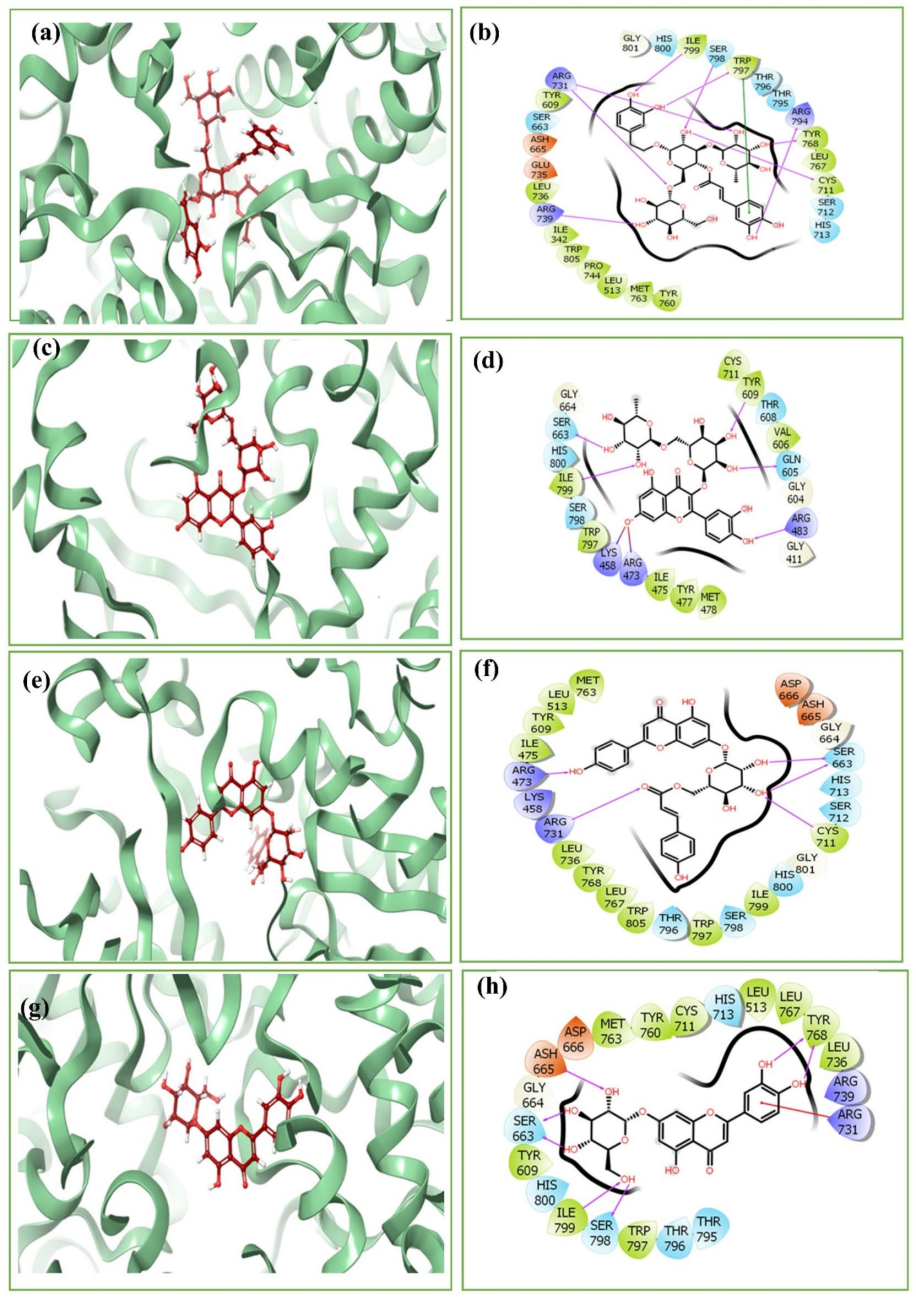
3D and 2D interaction diagrams of the docked RDRP-phytochemical complex i.e. (**a**-**b**) RDRP-echinacoside, (**c**-**d**) RDRP-rutin, (**e**-**f**) RDRP-echinacin, (**g**-**h**) RDRP-cynaroside.

Several significant interactions that contribute to the selected compound’s high binding affinities were identified by analyzing the molecular interactions between them and the RDRP of the Zika virus. With several crucial amino acid residues (like Arg^731^, Arg^739^, Ile^799^, Ser^798^, Trp^797^, Arg^794^, Tyr^768^, and Cys^711^) in the binding site, echinacoside created seven hydrogen bonds with the binding site of the viral protein and pi-pi stacking interaction was also displayed by echinacoside compound with Arg^794^ residue of the protein. Amino acid residues Ser^663^, Ile^799^, Tyr^609^, Gln^605^, and Arg^483^, rutin demonstrated five hydrogen bond interactions, and residues Lys^458^ and Arg^473^ demonstrated salt bridge formations that strengthened its binding. Echinacin produced five hydrogen bonds with Arg^473^, Arg^731^, Ser^663^, and Cys^711^. In contrast, Cynaroside showed seven hydrogen bond interactions with Ser^798^, Ile^799^, Ser^663^, Ash^665^, and Tyr^668^. Strong binding and complicated stabilization of the docked phytochemical complexes were made possible due to the formation of these hydrogen bonds. However, the reference molecule (G8O-G8L) demonstrated hydrogen bond interactions with only two residues (Trp^797^ and Arg^731^) of its binding site. Along with the hydrogen bond, several other interactions were also formed in each protein-ligand complex and are listed accordingly in (Table 1). These findings highlight the diverse molecular interactions involved in the binding of these compounds to the ZIKV RDRP, with each compound utilizing distinct hydrogen bonding patterns to achieve strong and specific binding (Fig. 2, Supplementary Fig. 2).

**Table 1:** List of RDRP protein residues participating in intermolecular interactions with phytochemicals.

Complex	H-bond	Hydrophobic	Polar	Salt bridge	π-π/*π-cation	Positive	Negative	Glycine
RDRP-echinacoside	Arg^731^(2), Arg^739^, Ile^799^, Ser^798^, Trp^797^, Arg^794^, Tyr^768^, Cys^711^	Tyr^609^, Leu^736^, Ile^342^, Trp^805^, Pro^744^, Leu^513^, Met^763^, Tyr^760^, Ile^799^, Trp^797^, Tyr^768^, Leu^767^, Cys^711^	Ser^663^, His^800^, Ser^798^, Thr^796^, Thr^795^, Ser^712^, His^713^		Trp^797^	Arg^731^, Arg^739^, Arg^794^	Ash^665^, Glu^735^	Gly^801^
RDRP-rutin	Ser^663^, Ile^799^, Tyr^609^, Gln^605^, Arg^483^	Ile^799^, Trp^797^, Ile^475^, Tyr^477^, Met^478^, Cys^711^, Tyr^609^, Val^606^	Ser^663^, His^800^, Ser^798^, Thr^608^, Gln^605^	Lys^458^, Arg^473^		Lys^458^, Arg^473^, Arg^483^		Gly^664^, Gly^604^, Gly^411^
RDRP-echinacin	Arg^473^, Arg^731^, Ser^663^(2), Cys^711^	Met^763^, Leu^513^, Tyr^609^, Ile^475^, Leu^736^, Tyr^768^, Leu^767^, Trp^805^, Trp^797^, Ile^799^, Cys^711^	Thr^796^, Ser^798^, His^800^, Ser^712^, His^713^, Ser^663^			Arg^473^, Lys^458^, Arg^731^	Asp^666^, Ash^665^	Gly^664^, Gly^801^
RDRP-cynaroside	Ser^798^, Ile^799^, Ser^663^(2), Ash^665^, Tyr^668^(2)	Trp^797^, Ile^799^, Tyr^609^, Met^763^, Tyr^760^, Cys^711^, Leu^513^, Leu^767^, Tyr^768^, Leu^736^	Thr^795^, Thr^796^, Ser^798^, His^800^, Ser^663^, His^713^		Arg^731^	Arg^731^, Arg^739^	Ash^665^, Asp^666^	Gly^664^
RDRP-G8O-G8L	**Trp**^**797**^, **Arg**^**731**^	**Ile**^**342**^, **Trp**^**797**^, **Met**^**763**^, **Tyr**^**760**^, **Leu**^**736**^, **Tyr**^**768**^, **Leu**^**767**^, **Trp**^**805**^, **Leu**^**513**^, **Leu**^**516**^, **Cys**^**711**^	**His**^**800**^, **Ser**^**798**^, **Thr**^**796**^, **Thr**^**795**^, **His**^**713**^, **Ser**^**712**^		**Arg** ^**731**^	**Arg** ^**739**^	**Glu** ^**735**^	**Gly** ^**801**^

Overall, the molecular interaction and re-docking analyses supported the highly potent binding affinities of echinacoside, rutin, echinacin, and cynaroside to the RDRP of the ZIKV. These substances displayed advantageous hydrogen bonds and other contacts inside the reference ligand binding site, indicating their potential as strong protein-targeting ZIKV replication inhibitors. Further, to confirm and investigate their binding stability, MD simulation experiments were performed.

### Molecular dynamics simulation

**Fig. 3:**
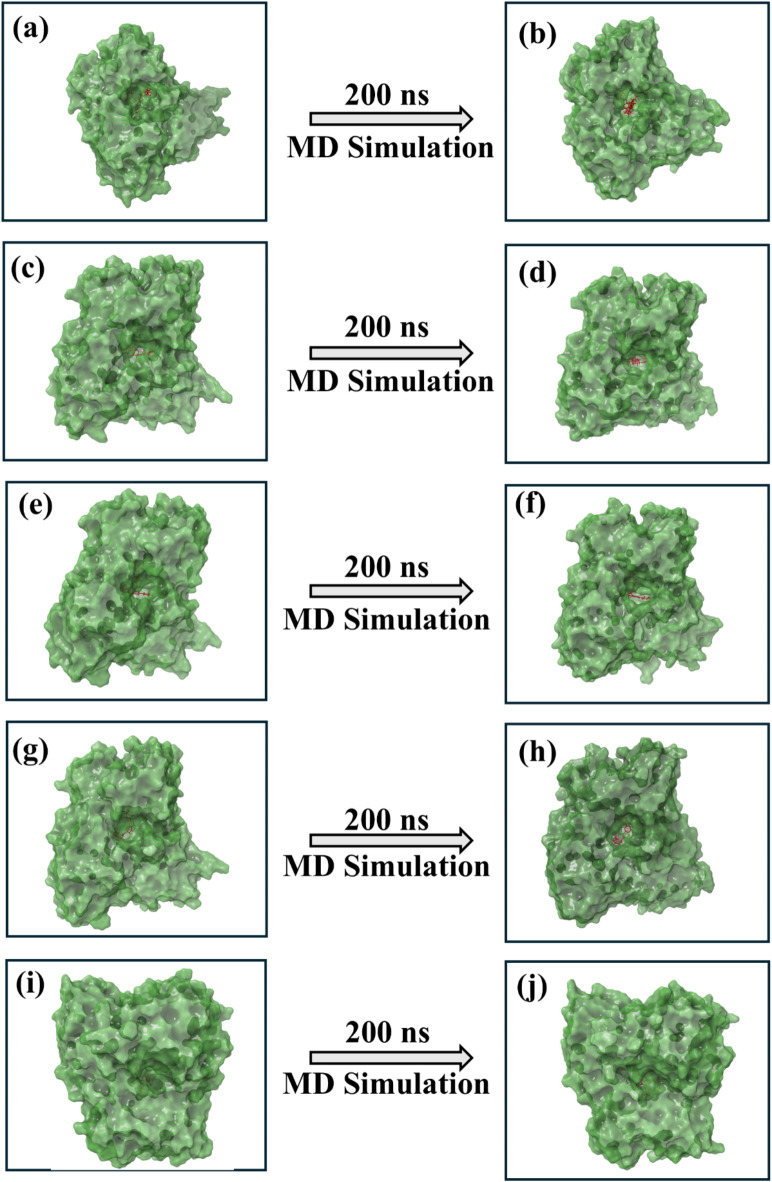
The 3D structure of the first and last pose of the docked RDRP-phytochemical compounds i.e., (**a**-**b**) RDRP-Echinacoside (**c**-**d**) RDRP-Rutin, (**e**-**f**) RDRP-Echinacin, (**g**-**h**) RDP-Cynaroside (**i**-**j**) Control.

Molecular dynamics simulations were conducted to investigate the protein-ligand complexes of echinacoside, rutin, echinacin, cynaroside, and the reference molecule with the Zika virus RDRP. The root-mean-square deviation (RMSD) analysis was performed to assess the stability and conformational changes of the complexes throughout the simulation. The RMSF measures how much an atom or group of atoms moves compared to a fixed structure. It calculates the average displacement for all the atoms. An in-depth understanding of the structural conformational change of protein structure and binding and displacement pattern of the ligand molecule in the protein-ligand complex that takes place during a continuous series of dynamic motions till the end of a particular time frame can be possible through MD simulation analysis. This can be possible by generating the three-dimensional structure of the initial pose and final pose of the protein-ligand complex retrieved from the 200 ns simulation trajectory of each complex. In this study, during the structural analysis of the initial and final pose of the RDRP-phytochemical complex, it was observed that the RDRP protein in each complex, including the reference complex shows, exhibited a very minimum amount of structural conformational changes during the simulation, and the phytochemicals showed acceptable displacement by remaining bound to the protein’s binding site. These three-dimensional structural observations help to conclude that the protein-ligand complex remains in a stable state during the period of MD simulation and may have the ability to inhibit the function of viral protein compared to the reference complex (Fig. 3). To further confirm these findings, the statistical analysis that includes the RMSD, RMSF, and molecular contacts formed in each RDRP-phytochemical complex was studied by retrieving the necessary data from the simulation trajectories.

### RMSD analysis

**Fig. 4:**
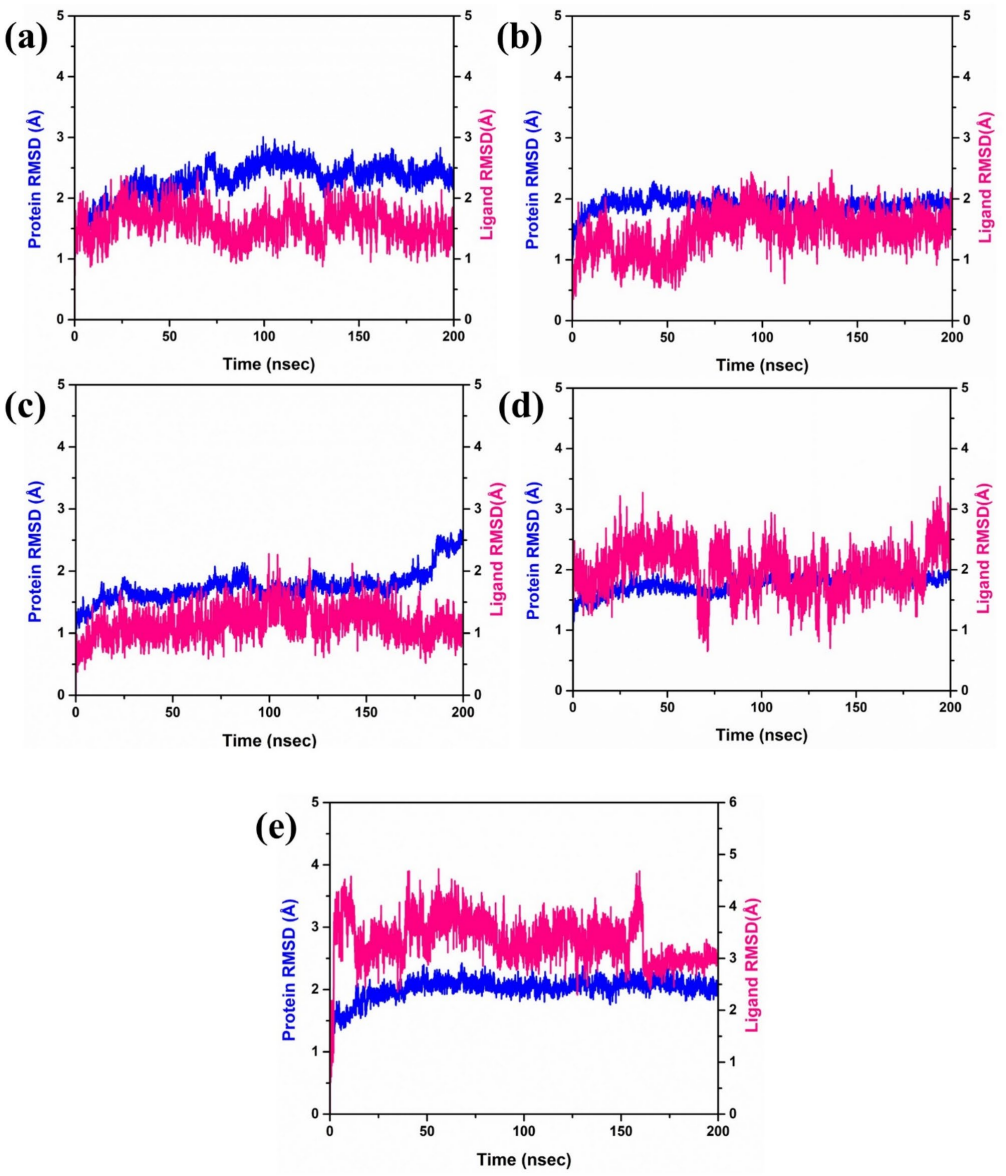
RMSD plot of the docked RDRP-phytochemical compounds i.e., (**a**) RDRP-Echinacoside (**b**) RDRP-Rutin, (**c**) RDRP-Echinacin, (**d**) RDP-Cynaroside and, (**e**) Control extracted from their 200 ns simulation trajectories.

Studied complexes were further analyzed using Root Mean Square Deviation (RMSD) and indicated different patterns of stability for protein as well as the ligand component as shown in **(**Fig. 4). Protein RMSD of complex 1 was found to be around 2.5 Å and RMSD of ligand was between 1 and 2 Å, implies moderate stability with slight feasibility in the position of ligand. On the other hand, Complex 2 had a slightly more stable structure with RMSD of 2 Å for the protein and the ligand also oscillated marginally between 1 and 2 Å indicating a stable protein-ligand complex. In Complex 3, the protein remained stable where the RMSD is less than 2 A throughout duration of 180ns. However, a prominent raise of RMSD more than 2.5 Å was observed in the range of 180–200 ns, which might point to the structural flexibility or conformational changes in the protein. In this case, the stability was maintained at a relatively low fluctuation range of 1 to 1.5 Å for the RMSD of the ligand. On the other hand, another complex maintained stable protein RMSD less than 2 Å, while ligand RMSD fluctuated between 2 and 3 Å. This implies that though the protein conformation did not change much during molecular dynamics simulation, the ligand molecule was significantly flexible. For the control complex, the protein kept an RMSD of 2.5 Å, while the ligand exhibited more significant deviations, with RMSD values ranging from 2.5 to 4.5 Å up to 160 ns. In 160 ns MD simulation, the ligand RMSD rose higher than 3 Å, suggesting that the stability was affected, or the conformation has changed.

### RMSF analysis

**Fig. 5:**
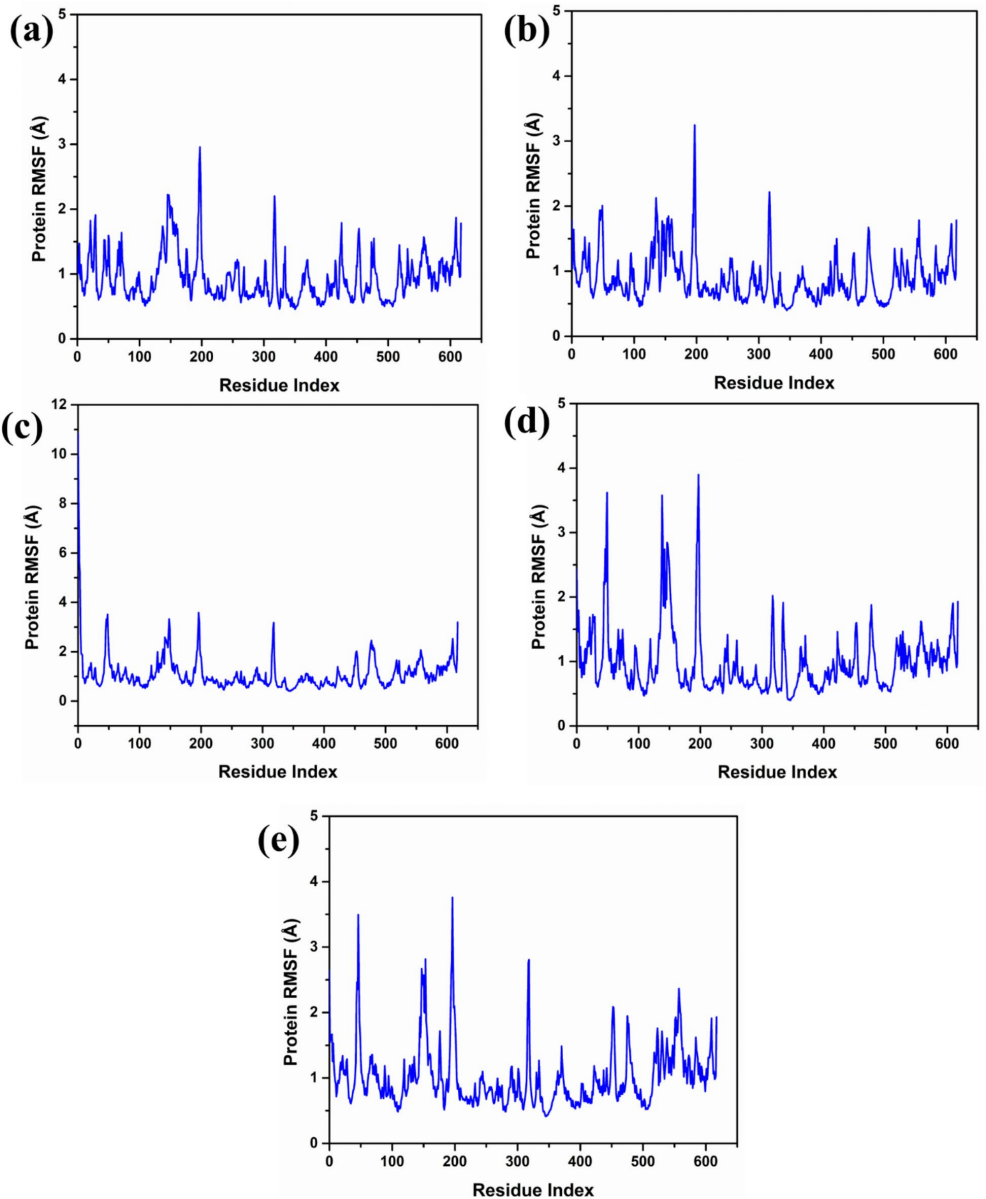
RMSF plot of the docked RDRP-phytochemical compounds i.e., (**a**) RDRP-Echinacoside (**b**) RDRP-Rutin, (**c**) RDRP-Echinacin, (**d**) RDP-Cynaroside and, (**e**) Control extracted from their 200 ns simulation trajectories.

The root mean square fluctuation (RMSF) values for the protein residues from 190 to 210 are shown to exhibit different flexibility patterns for the protein residues in various complexes. In complex 1, this residue has shown an RMSF value less than 3 Å confirming moderate flexibility in the backbone of protein. Interestingly, Complex 2 exhibited a slightly higher RMSF value of 3.5 Å for the same residues range which pointed out higher fluctuations. A Complex 3 had an RMSF value of less than 4 Å in residue 190–210, this implies that although flexibility occurred in the protein, its stability remained high. Likewise, Complex 4 had RMSF value of 4 Å within the same range of residues revealing similar degree of fluctuations. On the other hand, the control complex had an RMSF less than 4 Å for residues 190–210 similar to the range of values observed for the experimental complexes and hence similar flexibility patterns for the tested complex. Based on this analysis, all the complexes exhibit moderate variations of the protein in terms of its mobility within this range of residues. The RMSF analysis is shown in (Fig. 5).

### Molecular contact analysis of protein-ligand complexes from MD trajectories

**Fig. 6:**
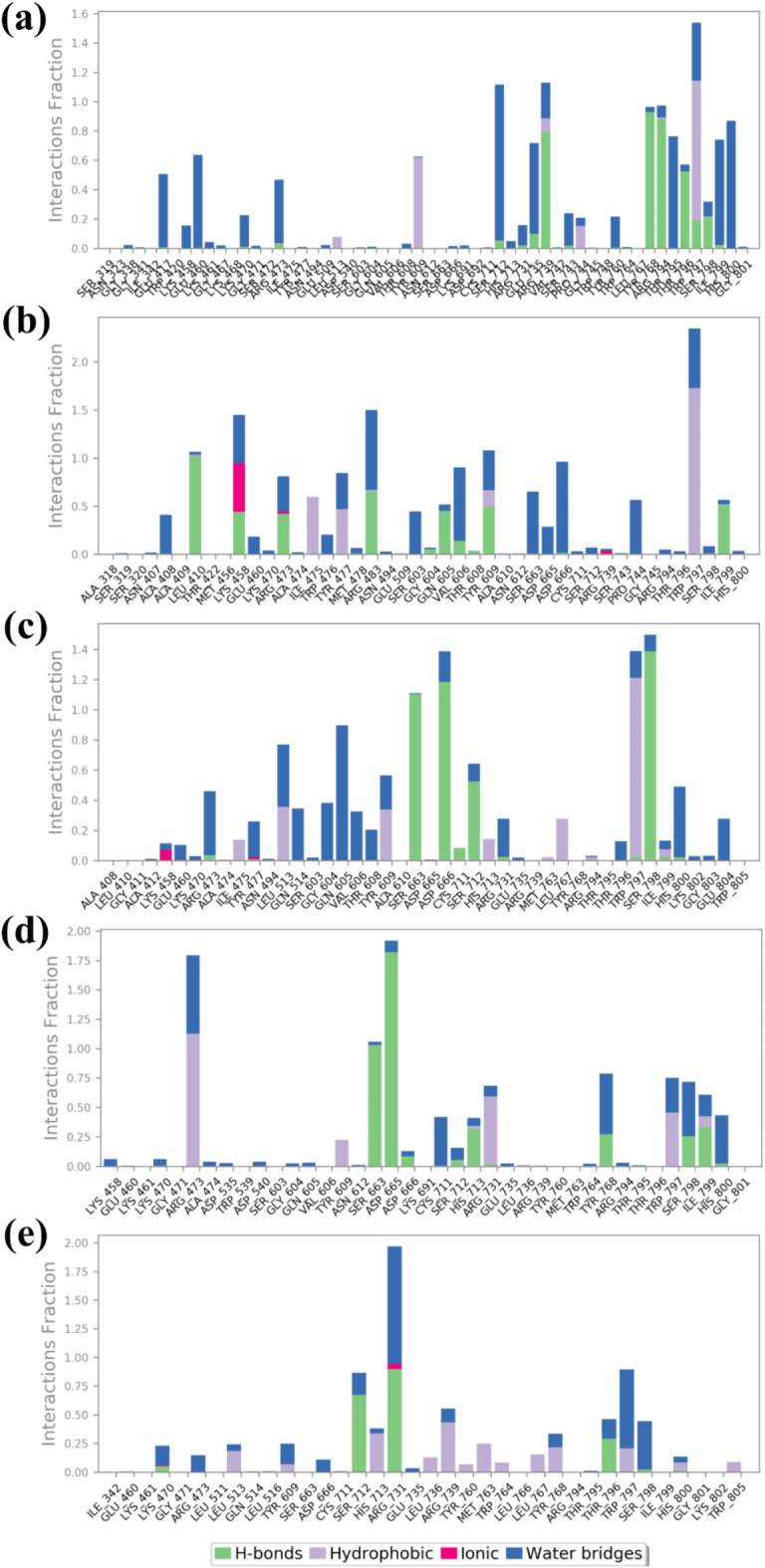
The graphical representation of molecular contact analysis of the RDRP-phytochemical compounds (**a**) RDRP-Echinacoside (**b**) RDRP-Rutin, (**c**) RDRP-Echinacin, (**d**) RDP-Cynaroside and (**e**) control extracted from their 200 ns simulation trajectories.

**Fig. 7:**
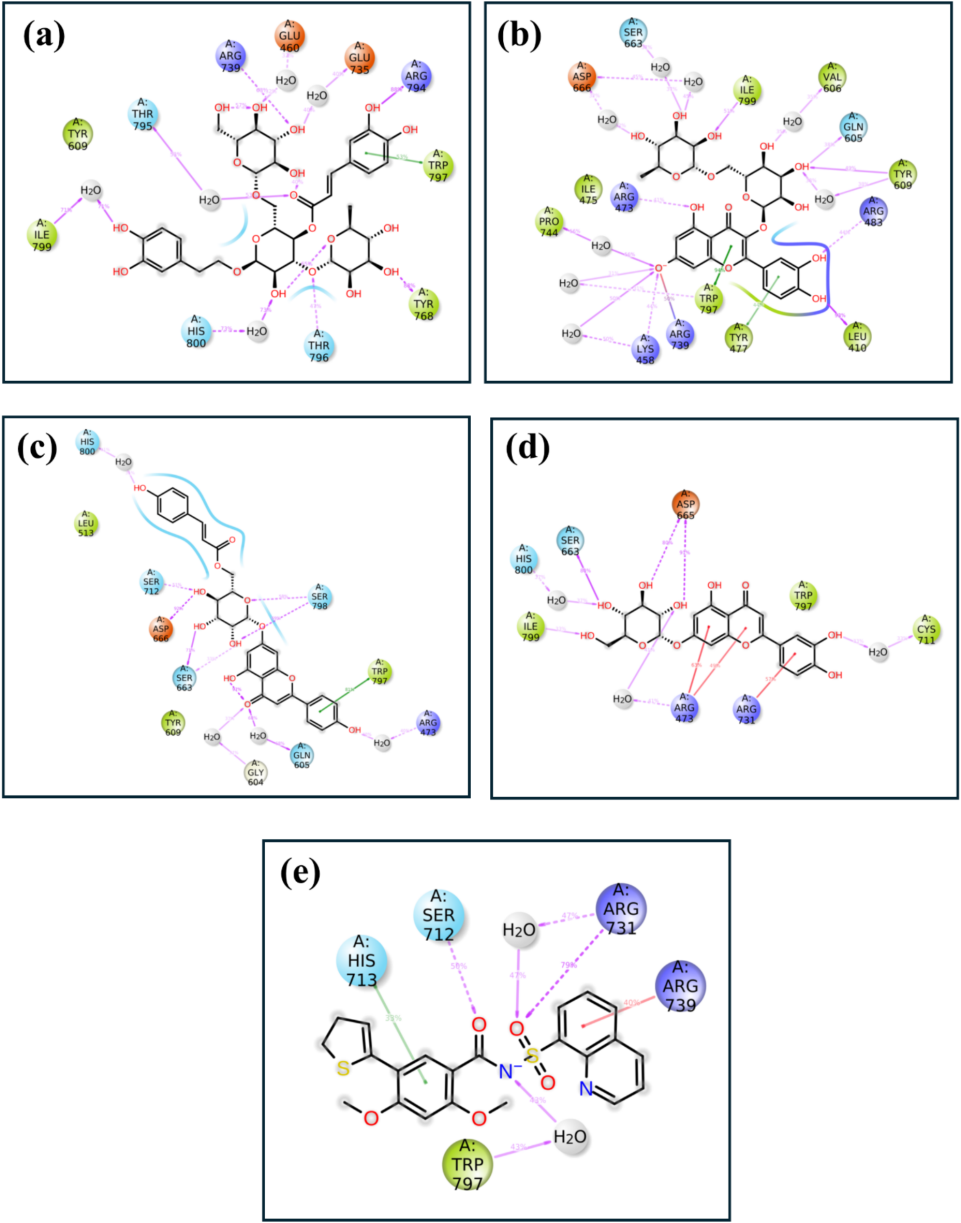
2D analysis of molecular contact analysis of the RDRP-phytochemical compounds (**a**) RDRP-Echinacoside (**b**) RDRP-Rutin, (**c**) RDRP-Echinacin, (**d**) RDP-Cynaroside and (**e**) control extracted from their 200 ns simulation trajectories.

The molecular contact analysis of the RDRP-phytochemical compounds focuses on finding the major interaction, such as hydrogen bonds, hydrophobic bonds, and ionic bonds, along with water bridge formation formed between the protein-ligand complex during the MD simulation, as shown in (Figs. 6 and 7). These are retrieved from the simulation trajectory of each docked complex, and the significant bonds formed with the protein residues for the maximum amount of time during the simulation period are observed to analyse the stability of each docked complex during MD simulation. So, in the RDRP- Echinacoside complex, mostly hydrogen bonds and water bridges are formed for more than 50% of the simulation period. Trp^797^ residue contributes to both hydrogen formation of 200 ns simulation time. Also, Ile^799^ participates in water bridge formation for 50% of the simulation time. Likewise, in the RDRP- Rutin complex, Trp^797^, and Tyr^477^ residues are responsible for the hydrogen bond formation 50–100% of the total interaction fraction, while Pro^744^, Tyr^609^ residue contributes to hydrophobic bond and water bridge formation for more than 50% of the simulation time. Similarly, in RDRP- Echinacin, namely Ser^798^, Asp^666^ and Ser^663,^ form hydrogen bonds for 100% of the total simulation period. The residue Trp^797^ contributes to hydrophobic bond for more than 80% of the overall simulation period. Gly^604^ residue participates in water bridge formation for 40% of the simulation time in this complex. Also, in RDRP- Cynaroside complex, Ile^799^ hydrophobic interact more than 30% during simulation. The residues also contribute to bond formations, but the mentioned residues play a significant role in the stabilisation of the complex. Moreover, in the reference complex residues, His^713^ contributed to hydrogen bond formation for 30% of the total simulation period, whereas Trp^797^ formed hydrophobic and water bridge interaction for more than 40% of the total simulation period.

### MM/GBSA analysis

**Fig. 8:**
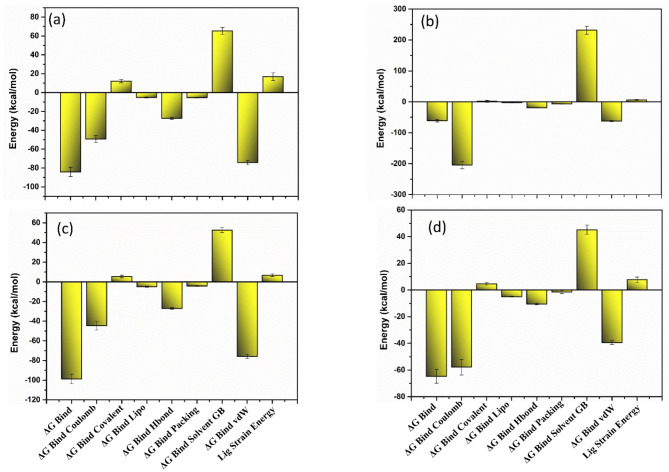
The graphical representation of the free binding energy of the RDRP-phytochemical compounds (**a**) RDRP-Echinacoside (**b**) RDRP-Rutin, (**c**) RDRP-Echinacin, and (**d**) RDP-Cynaroside calculated using the MM/GBSA method.

MM/GBSA analysis is a computational technique employed to calculate the free binding energy between a ligand and its target protein or receptor. It combines a molecular mechanics force field with a generalized Born or surface area-based solvation model to account for the intermolecular interactions and solvation effects. For estimating the free binding energy, the conformations extracted from the last 50 ns of the 200 ns MD simulation trajectories were utilized. The free binding energy is calculated by the total energy contribution of all the intermolecular interactions. For a better understanding of the individual contributions of these intermolecular interactions, such as covalent, hydrogen binding, Vander Waal etc., were also calculated. This helps to understand the significant role of each energy dissociation component in the stabilisation of each RDRP-phytochemical complex. The analysis of the overall binding free energy for the protein docked with the screened phytochemical compounds of *E. angustifolia* revealed significant energy values compared to the corresponding reference complexes. RDRP- Echinacin complex exhibited maximum binding free energy (ΔG_Bind_) value of −98.77 kcal/mol, followed by RDRP- Echinacoside (−84.13 kcal/mol), RDRP- Cynaroside (−64.65 kcal/mol) and RdRp- Rutin (−61.46 kcal/mol). However, the reference complex exhibited a binding energy of −50.18 kcal/mol, which is less than the selected phytochemical complex. Also, in the energy dissociation component analysis, it was seen that ΔG_Bind Coulomb_ and ΔG_Bind vdW_ contribute maximum energy in the stabilisation of the RDRP-phytochemical complex. The free binding energy analysis finalises that the selected phytochemical compounds tend to stabilise the complex and may participate in inhibiting the viral protein **(**Fig. 8, Supplementary Fig. 3, and Table S2).

### Principal component analysis (PCA)

**Fig. 9:**
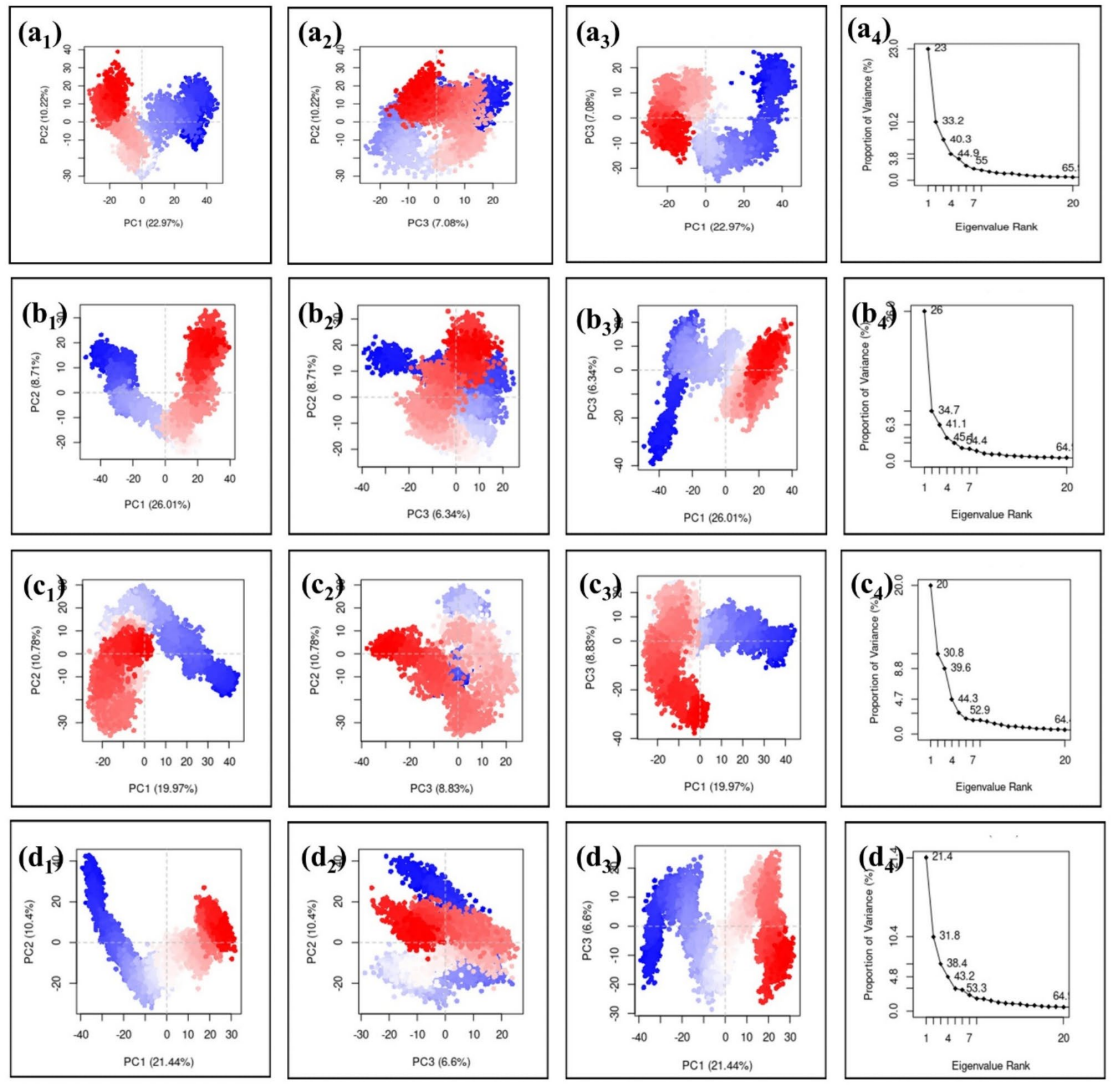
Cluster analysis and scree plot obtained for the RDRP-phytochemical compounds (**a**) RDRP-Echinacoside (**b**) RDRP-Rutin, (**c**) RDRP-Echinacin, and (**d**) RDP-Cynaroside during PCA analysis.

PCA is a statistical technique used to identify the main modes of motion and highlight the most significant variations within the system. By performing PCA on the MD simulation trajectories spanning 200 ns, valuable insights were gained regarding the RDRP- phytochemical complex. The analysis demonstrated that the first three principal components, PC1, PC2, and PC3, accounted for a significant amount of the total variance observed in the dataset. Visualizing the results of PCA by generating a cluster plot revealed different clustering patterns of the poses generated during the simulation by each docked complex, indicating the presence of conformational dynamics induced by the phytochemical compounds. This suggests that the phytochemical compounds have the potential to induce different structural changes in the protein. The sum total of the first principal component (PC1 + PC2 + PC3) of each RDRP-phytochemical complexe i.e., RDRP- Echinacoside, RDRP- Rutin, RDRP- Echinacin, RDRP- Cynaroside, and reference complex poses, RDRP-G8O-G8L (Reference ligand) are 40.27, 40.62, 39.58, 38.44 and 35.80% respectively This implies that the RDRP- Echinacoside complex may exhibit maximum stability due to the presence of similar conformers compared to all other phytochemicals and reference molecule. The cluster analysis of these complexes and reference molecule states that all the complexes are stable as the clusters are mostly overlapped with minimum splitting, and the scree plot analysis of these complexes also shows that the plotting of eigenvalues is in a curve slope rather than steep slope elbow formation. These findings suggest that all the docked complexes are stable. So, according to the results obtained from PCA analysis, all the complexes display acceptable stability compared to the reference complex (Fig. 9 and Supplementary Fig. 4).

### Dynamic cross corelation analysis (DCCM)

**Fig. 10:**
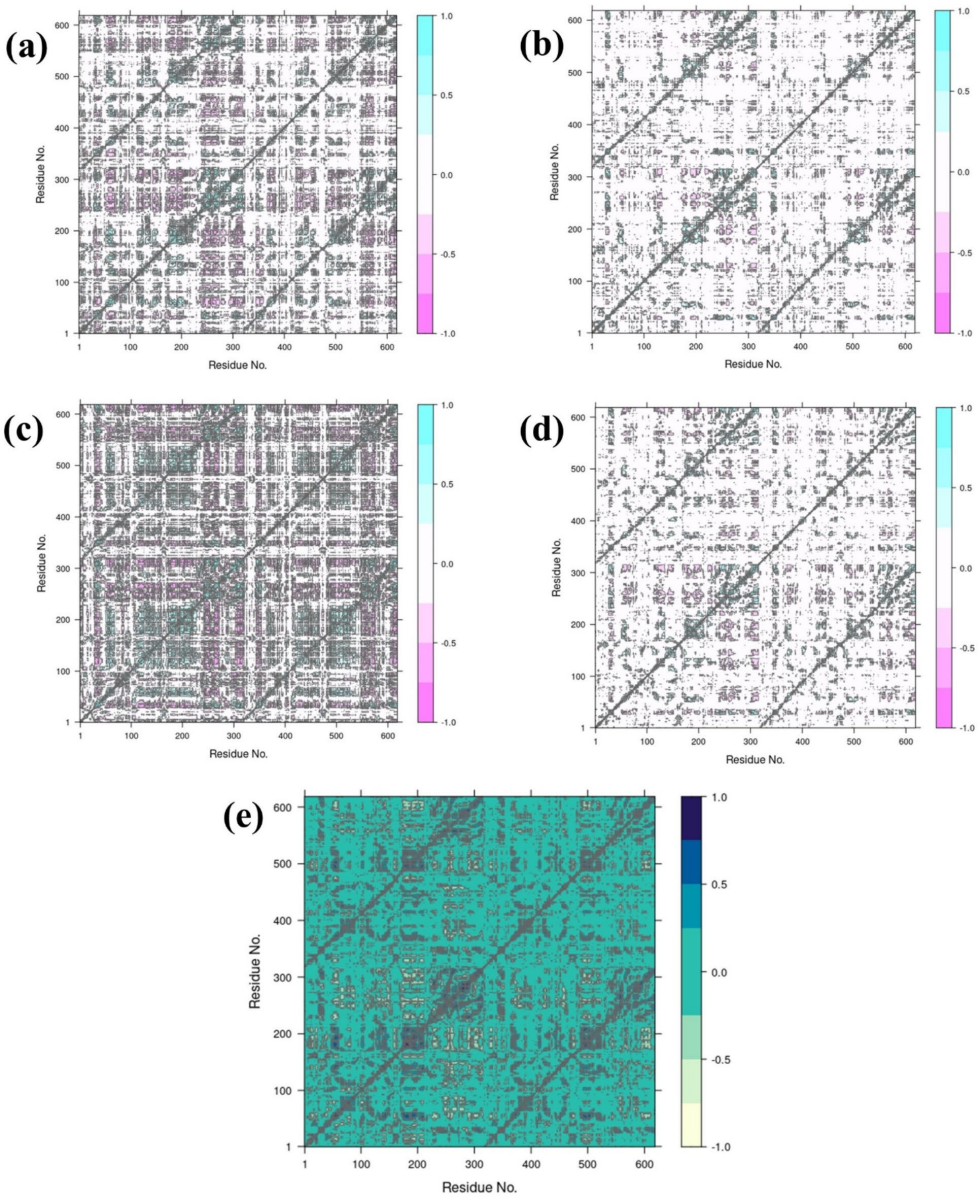
DCCM analysis of RDRP-phytochemical compounds such as (**a**) RDRP-Echinacoside (**b**) RDRP-Rutin, (**c**) RDRP-Echinacin, (**d**) RDP-Cynaroside and (**e**) control.

To determine how each of the four compounds affects the protein’s dynamic interactions relative to the control, the DCCM analysis of the dynamic cross-correlation matrix (DCCM) is in (Fig. 10). In the control (e), there are strong and frequent motions, which suggests that the protein showed extensive coordinated movements. This allows comparing how each compound alters the proteins’ inherent flexibility and motion. Compound Echinacoside displays the positive shift in residues and has variable coupling motion to harmonize the fluctuation to other residues but with less negative coupling motion. Such flexibility may compromise the function of the protein; while being more flexible than the constant structure, it may compromise the stability of the protein. Compound Rutin, on the other hand exhibits relatively fewer regions of high values of correlation coefficient and may be implying fewer dynamic interactions and possibly leads to a stiffness of the protein. This lowered flexibility could help strengthen structure rigidity of the protein, thus it has lesser conformational changes which can be useful in engineering more stable enzymes. Compound Echinacin shows highly anti-correlated motions and therefore, the specific fragments of the protein are moving in the opposite directions, indicating structural changes due to this compound. This could affect active or flexible sites of the protein which may be of particular importance for change in enzyme function or stability. The results for compound Cynaroside are lower, indicating a moderate flexibility and dynamic coordination and positive as well as negative dynamic correlations. This balance might help the protein for some of the loops to remain as flexible as before and at the same time it can fix some regions which is very important for increasing substrate specificity or increasing the turnover number.

### Secondary structure analysis

**Fig. 11:**
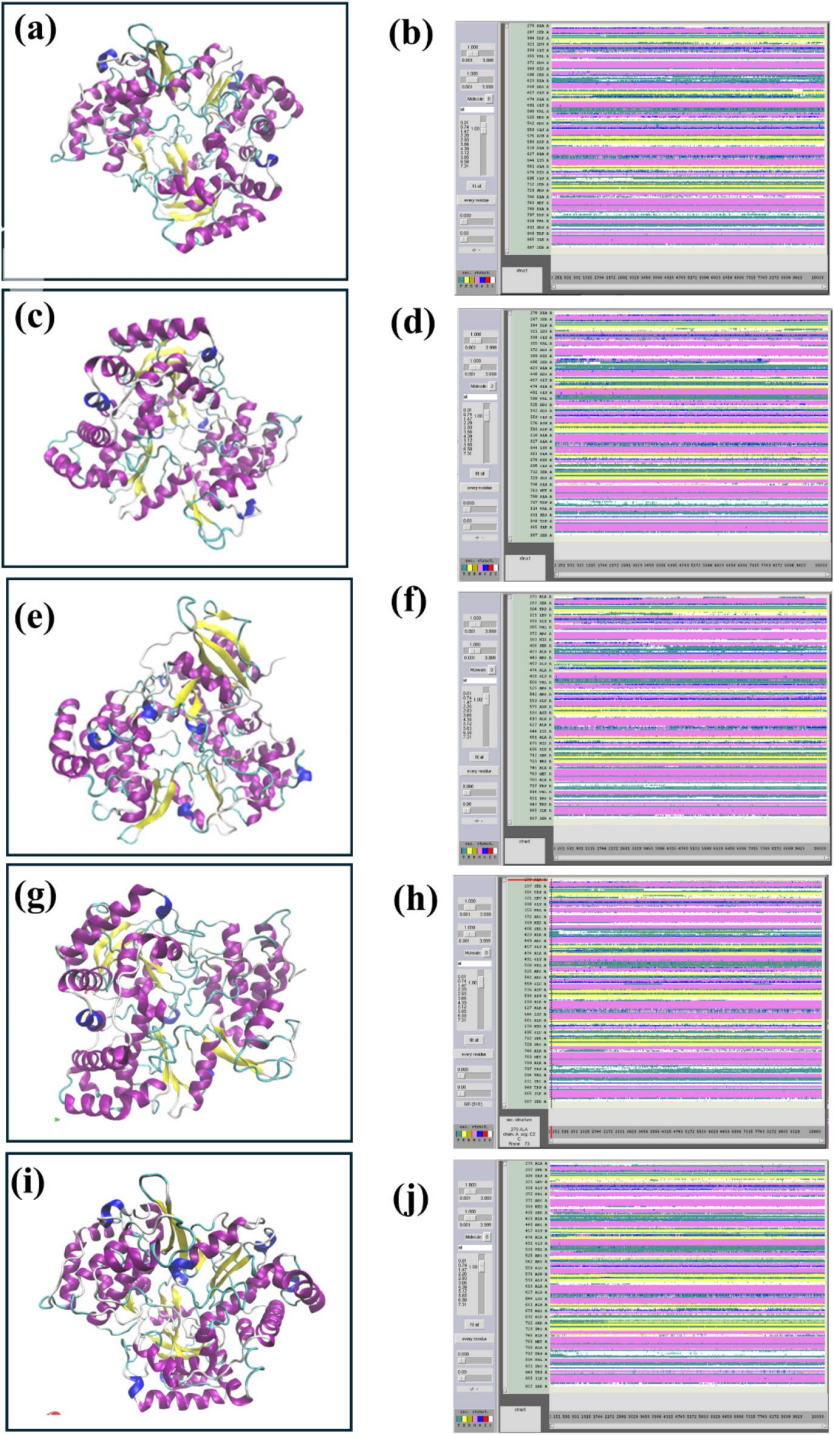
Secondary structure analysis of RDRP-phytochemical compounds such as (**a**-**b**) RDRP-Echinacoside (**c**-**d**) RDRP-Rutin, (**e**-**f**) RDRP-Echinacin, and (**g**-**h**) RDP-Cynaroside and (**i**-**j**) control.

The secondary structure analysis is shown in (Fig. 11). In the complex second protein in complex 1 showed structure predominantly alpha-helical conformation with some beta-strand regions interspersed throughout. Loops are connecting these secondary structures. Since the compound interacts mainly with loop regions, it may cause localized flexibility, but the overall protein stability seems unaffected. The secondary structure of the protein in the complex 2 also maintains a strong alpha-helical structure, but the loops appear slightly more extended, with the beta strands still present in similar regions. The compound might induce slight conformational shifts near loop-helical junctions, potentially influencing protein activity by altering surface accessibility. The secondary structure analysis of complex 3 revealed that beta strands are more prominent compared to the first two structures with clear well-formed beta sheets. Alpha helices are still prevalent but seem slightly more compact. This indicates that binding could stabilize certain regions of the protein through interactions with beta strands, leading to potential structural constraints that might enhance or suppress the protein’s natural function. Secondary Structure of complex 4 exhibits a more balanced mix of alpha helices and beta sheet. The impact of this compound might be more on the protein’s functional dynamics rather than its structural integrity, potentially affecting interactions with other proteins or substrates and, with loops extending further from the core structure. The control secondary structure showed the native structure of the protein maintaining its natural secondary structure conformation. The alpha helices, beta strands, and loops are well-defined and follow the expected alignment pattern. The protein remains structurally sound without external influences, serving as a benchmark to measure the effects of the other four compounds.

### RG-RMSD based FEL

**Fig. 12:**
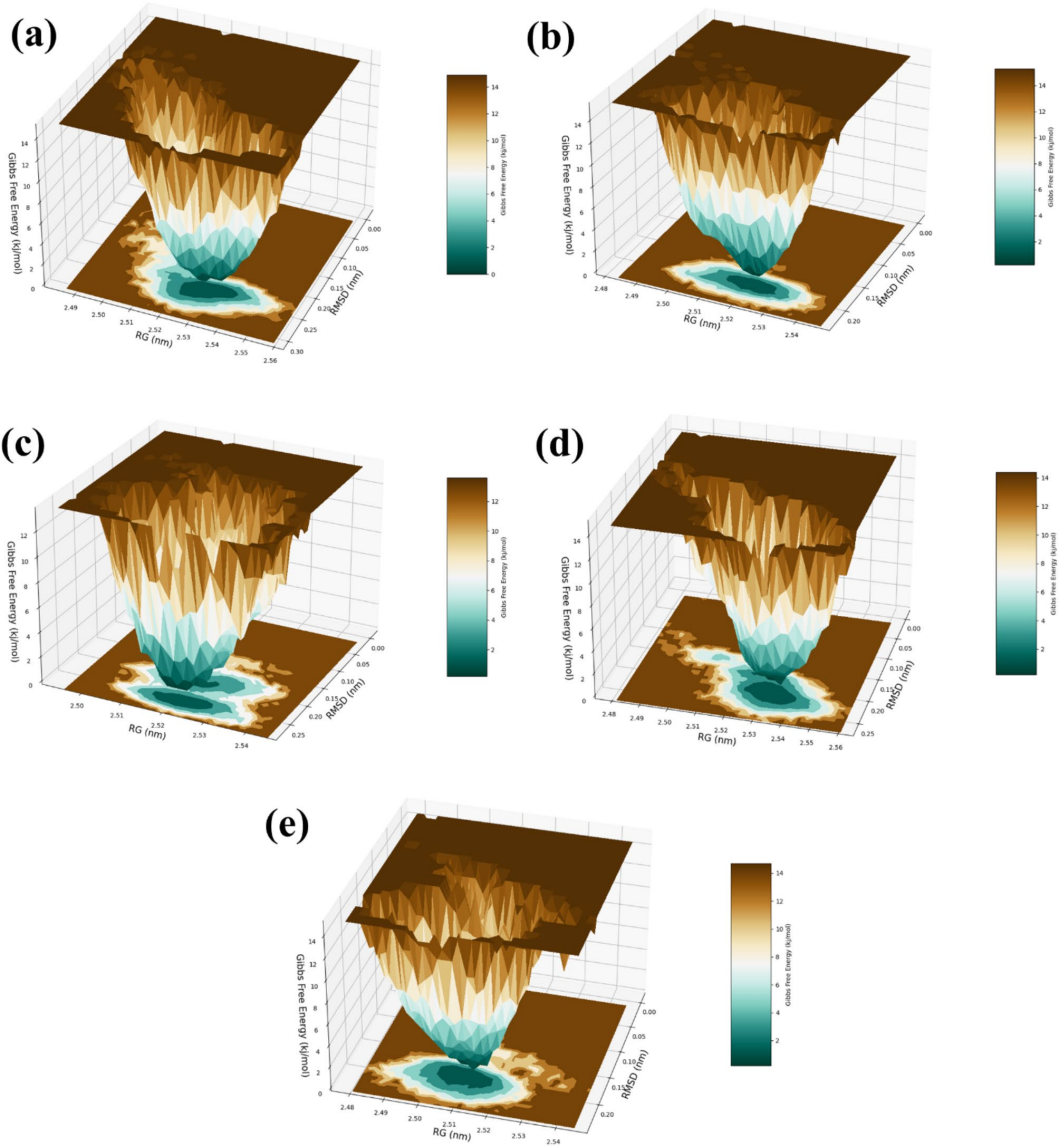
RG-RMSD based analysis of RDRP-phytochemical compounds such as (**a**) RDRP-Echinacoside (**b**) RDRP-Rutin, (**c**) RDRP-Echinacin, (**d**) RDP-Cynaroside and (**e**) control.

The RG-RMSD-based free energy landscape (FEL) analysis of the four complexes—RDRP-Echinacoside, RDRP-Rutin, RDRP-Echinacin, and RDRP-Cynaroside—and the control highlights key differences in energy transitions, stability, and the dynamic behavior of the protein-ligand interactions during molecular dynamics (MD) simulations as shown in (Fig. 12). For the RDRP-Echinacoside complex, the FEL plot reveals a highly stable lower energy state, indicating that the complex reaches a favorable conformation with minimal transitions to higher energy regions. This deep energy well signifies strong and stable interactions between the Echinacoside ligand and the RDRP protein. The lack of significant transitions to higher energy states suggests that the complex remains in a stable conformation throughout the simulation, allowing little conformational flexibility. This indicates tight binding and an effective interaction between the ligand and the protein. In the case of the RDRP-Rutin complex, the FEL shows a stable lower energy state, but the transitions to higher energy regions are slightly more gradual compared to RDRP-Echinacoside. This suggests that while the Rutin complex maintains a stable binding conformation, it allows for more flexibility within the protein-ligand complex. The energy landscape is more rugged, indicating the presence of alternative, less stable conformations that the system occasionally explores during the simulation. While still exhibiting stability, the Rutin complex allows for more dynamic behavior compared to Echinacoside. The RDRP-Echinacin complex demonstrates a broader lower energy state, reflecting a stable binding conformation but with more conformational diversity. The wider energy basin implies that the RDRP-Echinacin complex can adopt multiple conformations, which may lead to more flexibility in the system. This complex undergoes more frequent energy transitions, and higher energy states appear more often compared to RDRP-Echinacoside and RDRP-Rutin. This indicates that the interactions between Echinacin and the protein are slightly weaker, allowing for more conformational variability and less rigid binding. The RDRP-Cynaroside complex displays a well-defined lower energy state, similar to RDRP-Echinacoside. The sharp energy well indicates that the Cynaroside complex forms a strong, stable interaction with the RDRP protein, resulting in minimal conformational shifts. Like Echinacoside, Cynaroside restricts conformational flexibility, as the complex stays in a stable state throughout the simulation. The energy transitions are infrequent, and the appearance of higher energy states is sparse, which implies that Cynaroside effectively stabilizes the protein-ligand complex. In contrast, the control compound shows a shallow lower energy state with frequent transitions to higher energy regions. This broader energy landscape indicates that the control does not stabilize the protein-ligand complex as effectively as the test compounds. The frequent transitions suggest that the control complex samples a wide range of conformational states, reflecting weaker interactions and greater flexibility in the system. This indicates that the control compound does not achieve the same level of binding stability as the tested ligands.

## Conclusion

In conclusion, this computational study investigates potential bioactive compounds from *E. angustifolia* as inhibitors of the ZIKV RDRP, one of the most critical viral enzymes in the replication process. Employing a virtual screening approach, molecular docking, and dynamic simulations, four promising candidate compounds were identified: echinacoside, rutin, echinacin, and cynaroside. These compounds had good binding affinities, stable interactions with key RDRP residues, and robust binding free energies, probably indicative of their effectiveness against RDRP. Further, advanced analyses such as PCA, MM/GBSA, and secondary structure assessment have proved the stability and efficacy of these complexes in disrupting the function of RDRP. The findings from this study indicate the value of compounds from *E. angustifolia* as candidates for antiviral applications, which needs experimental confirmation at a clinical level. This study provides a foundation for future research into plant-derived antiviral therapies, emphasizing the importance of phytochemicals in addressing emerging infectious diseases like ZIKV.

## Electronic supplementary material

Below is the link to the electronic supplementary material.

Supplementary Material 1

## Data Availability

The datasets generated and/or analysed during the current study are available upon request from the corresponding author.
