# Supplementary material for: Exploring Echinacea angustifolia for anti-viral compounds against Zika virus RNA-dependent RNA polymerase: a computational study
Source: Sci Rep. 2025 Feb 3;15:4060. doi: 10.1038/s41598-025-88481-8 (PMC11790867; doi:10.1038/s41598-025-88481-8)
Supplement: Supplementary file 1 — Supplementary Material 1 [file 41598_2025_88481_MOESM1_ESM.docx]

**Supplementary information**

**Exploring *Echinacea angustifolia* for Anti-Viral Compounds Against Zika virus RNA-dependent RNA polymerase: A Computational Study**

Mai M. El-Daly^1,2^, Leena H. Bajrai^1,3^, Thamir A. Alandijany^1,2^, Isra M. Alsaady^1,2^, Hattan S. Gattan^1,2^, Meshari M. Alhamdan^4^, Vivek Dhar Dwivedi^5,6*^, Esam I. Azhar^1,2 *^

^1^ Special Infectious Agents Unit – BSL3, King Fahd Medical Research Center, King Abdulaziz University, Jeddah - 21362, Saudi Arabia.

^2^ Department of Medical Laboratory Sciences, Faculty of Applied Medical Sciences, King Abdulaziz University, 21362 Jeddah, Saudi Arabia.

^3­­­­^ Biochemistry Department, Faculty of Sciences, King Abdulaziz University, Jeddah - 21362, Saudi Arabia.

^4^ Family Medicine Department, Faculty of Medicine, King Abdulaziz University, Jeddah - 21589, Saudi Arabia.

^5^ Center for Global Health Research, Saveetha Institute of Medical and Technical Sciences, Saveetha Medical College and Hospitals, Saveetha University, Chennai, India

^6^ Bioinformatics Research Division, Quanta Calculus, Greater Noida, India.  ^­­^

**Correspondences:**

**Vivek Dhar Dwivedi**

ORCID:

E. mail: [vivek_bioinformatics@yahoo.com](mailto:vivek_bioinformatics@yahoo.com)

**Esam I. Azhar**

ORCID: <https://orcid.org/0000-0002-1736-181X>

E. mail: [eazhar@kau.edu.sa](mailto:eazhar@kau.edu.sa)

**Results and Discussion**

**S1.1 Structure-based virtual screening**

#### Table S1. List of Phytochemical compounds obtained after virtual screening against RNA-Dependent RNA Polymerase protein of Zika Virus.

| Compounds | Binding energy (Kcal/mol) |
| --- | --- |
| 5281771 | -10 |
| 5280805 | -9.6 |
| 6439941 | -9.4 |
| 5280637 | -9.3 |
| 5280794 | -9.1 |
| 6537500 | -8.9 |
| 44259796 | -8.7 |
| 5280343 | -8.2 |
| 1794427 | -8.2 |
| 5282102 | -8.1 |
| 5280443 | -7.8 |
| 5281654 | -7.6 |
| 5280863 | -7.5 |
| 5281515 | -7.4 |
| 91723653 | -7.3 |
| 14350 | -7 |
| 5988 | -6.9 |
| 93009 | -6.7 |
| 440946 | -6.7 |
| 11587035 | -6.7 |
| 6440397 | -6.6 |
| 11413953 | -6.4 |
| 16219508 | -6.1 |
| 1.32E+08 | -6.1 |
| 64685 | -6 |
| 6443006 | -5.8 |
| 149801 | -5.7 |
| 22041880 | -5.6 |
| 445639 | -5.5 |
| 22199 | -5.4 |
| 3931 | -5.3 |
| 985 | -5.3 |
| 10469 | -5.2 |
| 593849 | -5.2 |
| 11005 | -5.1 |
| 6441887 | -5.1 |
| 12811248 | -5 |
| 68972 | -5 |
| 5322026 | -4.9 |
| 441552 | -4.7 |
| 528755 | -4.6 |
| 25913 | -4.6 |
| 247 | -3.8 |
| 81653 | -3.6 |
| 6354 | -2.5 |

**S1.2. Re-docking and intermolecular interaction analysis**


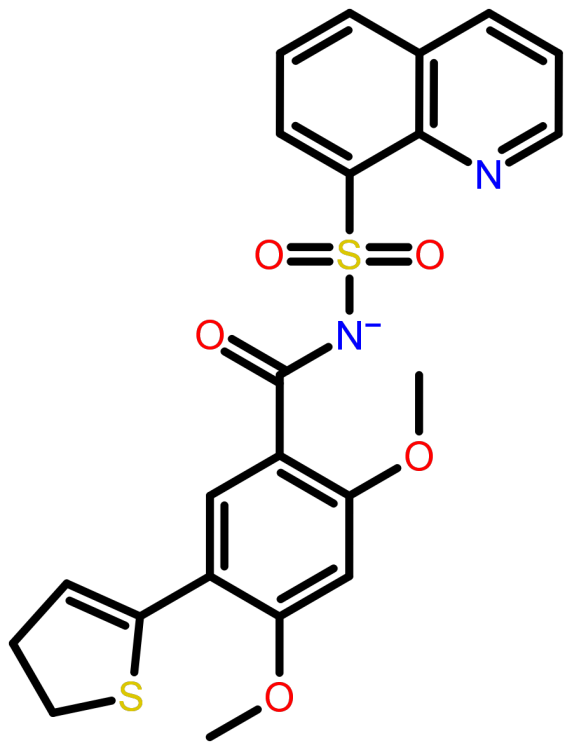


**Figure S1.** 2D structure of reference compound- G8O-G8L.


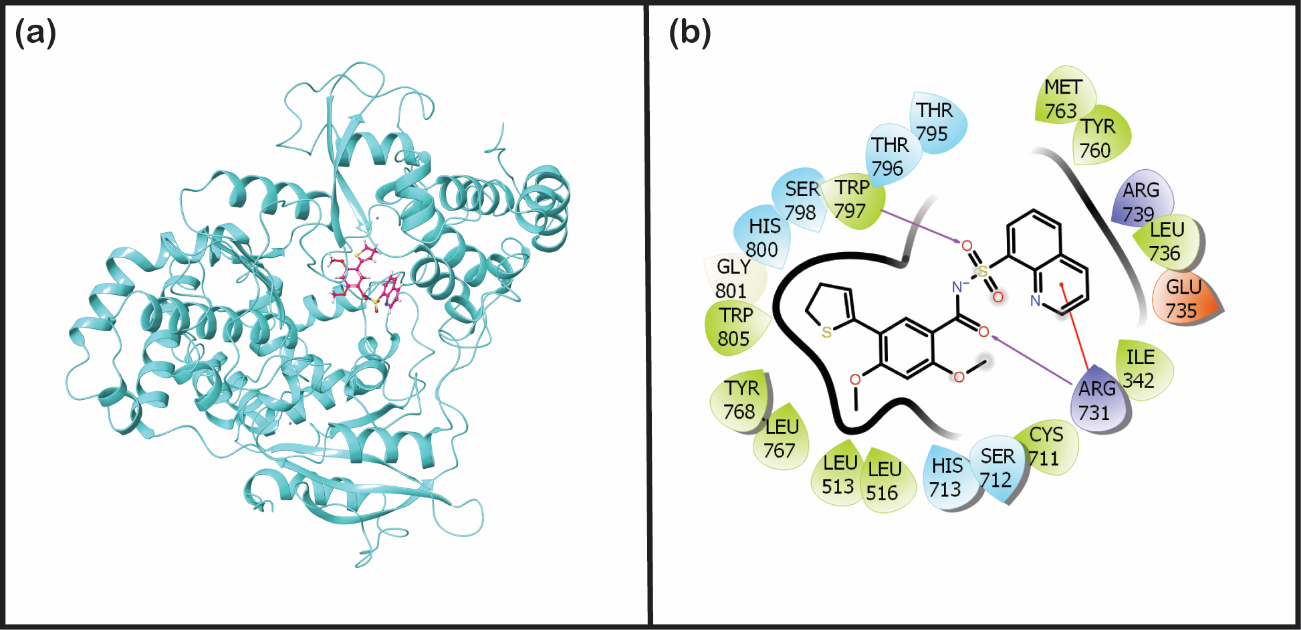


**Figure S2**. 3D and 2D docked complex poses of the reference complex.

**
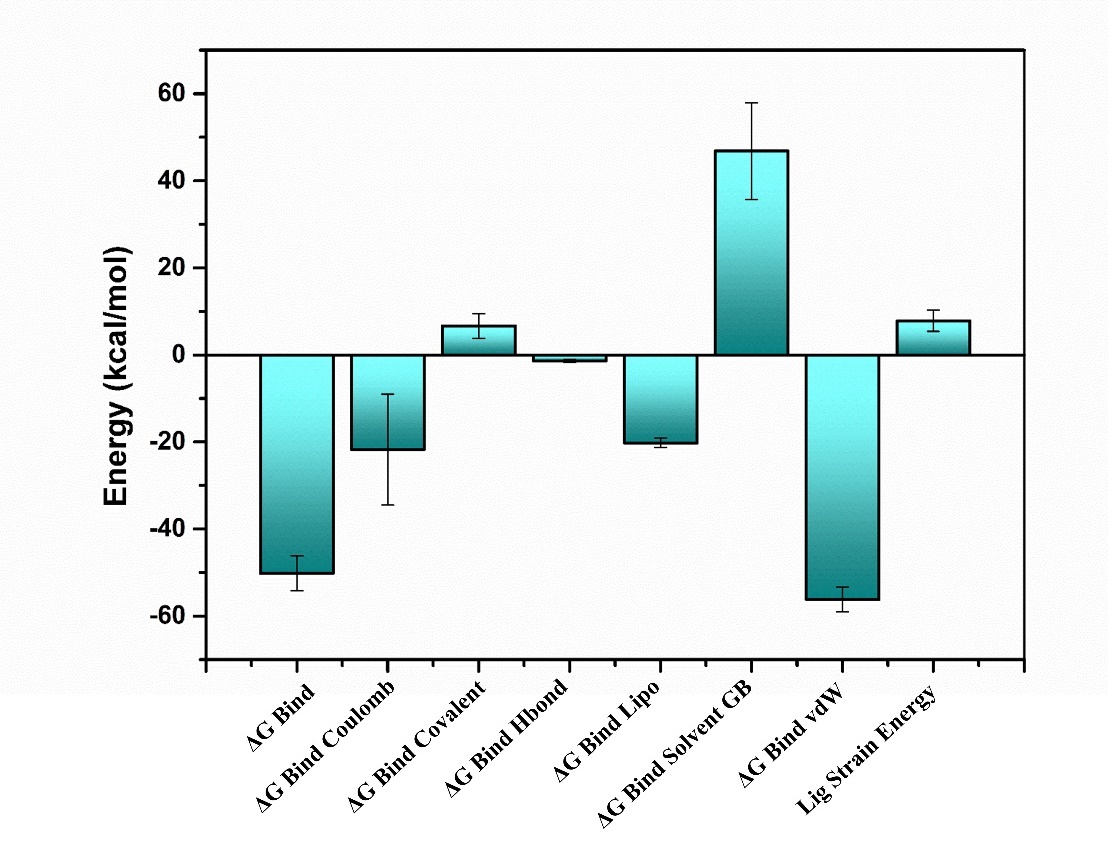
4.1 Endpoint Free binding energy calculation**

**Figure S3**. Calculated MM/GBSA analysis of the reference complex

| MM/GBSA components  (kcal/mol) | Echinacosides | Rutin | Echinacin | Cynaroside | G8O-G8L (reference) |
| --- | --- | --- | --- | --- | --- |
| ΔGBind | -84.13±4.96 | -61.46±3.76 | -98.77±4.83 | -64.65±5.16 | -50.18±4.03 |
| ΔGBind Coulomb | -49.23±3.62 | -204.22±11.93 | -44.53±4.31 | -57.81±5.87 | -21.76±12.76 |
| ΔGBind Covalent | 12.04±1.80 | 1.80±2.81 | 5.31±1.35 | 4.58±0.93 | 6.64±2.80 |
| ΔGBind Hbond | -5.30±0.57 | -2.48±0.42 | -4.96±0.62 | -5.04±0.26 | -1.39±0.31 |
| ΔGBind Lipo | -27.31±1.04 | -19.44±0.70 | -27.11±0.74 | -10.54±0.62 | -20.25±1.08 |
| ΔGBind Packing | -5.39±0.44 | -6.56±0.88 | -4.28±0.46 | -1.47±1.23 | -27.31±1.04 |
| ΔGBind Solv GB | 65.23±3.74 | 231.58±12.75 | 52.59±2.71 | 45.11±3.51 | 46.83±11.11 |
| ΔGBind vdW | -74.17±2.28 | -62.14±2.06 | -75.78±2.17 | -39.47±1.48 | -56.18±2.83 |
| Ligand Strain Energy | 16.86±4.10 | 5.82±1.75 | 6.60±1.47 | 7.69±1.98 | 7.83±2.42 |

**Table S2**- Binding free energy and energy disassociation components of each selected docked complexes calculated during the endpoint binding free energy calculation of each simulation trajectory.

**5. Principal component analysis**


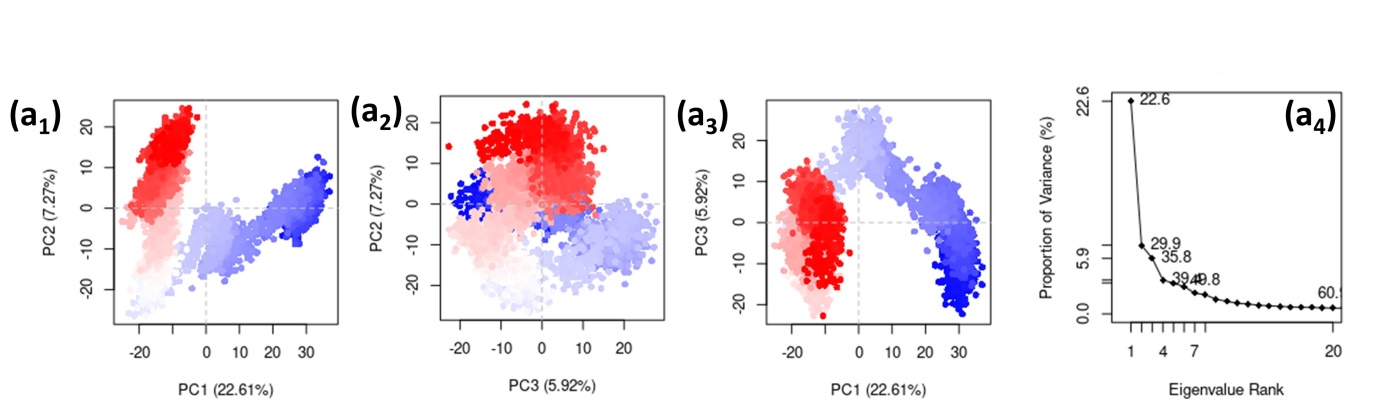


**Figure S4.** Principal component analysis for the molecular dynamic simulation trajectories of RNA-Dependent RNA Polymerase docked with reference compound. The percentage of total mean square displacement of residue positional variations recorded in each dimension is categorized by equivalent eigenvalue (PCs). The persistent color scale from blue to white to red directs the periodic jumps between the protein conformers duration the 100 ns simulation interval.
